# A pediatric case of atypical hemolytic uremic syndrome (aHUS): Could any infection play a triggering role? 

**DOI:** 10.5414/CNCS111209

**Published:** 2024-04-05

**Authors:** Nikolaos Gkiourtzis, Paraskevi Panagopoulou, Kyriaki Papadopoulou-Legbelou, Sofia Chantavaridou, Despoina Tramma

**Affiliations:** 4^th^ Department of Pediatrics, “Papageorgiou” General Hospital, Aristotle University of Thessaloniki, Greece

**Keywords:** atypical hemolytic uremic syndrome, influenza A, alternative complement pathway, children

## Abstract

A 12-year-old boy was transferred to our pediatric department from a rural hospital for fever, cough, and vomiting associated with thrombocytopenia, non-immune hemolytic anemia, and acute kidney injury, leading to the diagnosis of hemolytic uremic syndrome (HUS). A nasopharyngeal swab and a lower respiratory sample detected *Influenza A* by polymerase chain reaction (PCR). The patient was treated with oseltamivir and intravenous fluids in addition to fresh frozen plasma (FFP). Enteropathogenic* Escherichia coli *(EPEC) was detected in a stool sample by PCR. Serum antibodies for *Mycoplasma pneumoniae* (IgM and IgG) and *Helicobacter pylori* (IgA and IgG) were increased. Further work-up revealed elevated serum C5b-9 suggesting a simultaneous viral and bacterial infection-mediated complement overactivation leading to the diagnosis of atypical HUS (aHUS). An association between aHUS and influenza A is reported in the literature, but the correlation of EPEC, *Mycoplasma pneumoniae*, and *Helicobacter pylori* with aHUS is not well-established. Fresh frozen plasma was administered for a total of 3 days, followed by clinical and laboratory improvement. The patient has remained asymptomatic until the latest follow-up, 5 months after discharge. This case demonstrates the potential triggering role of different pathogens in aHUS pathogenesis to raise awareness in the pediatric community.

## Introduction 

Atypical hemolytic uremic syndrome (aHUS) is a rare type of thrombotic microangiopathy (TMA) that is characterized by microangiopathic hemolytic anemia, thrombocytopenia, and acute kidney injury without coexisting disease [1, 2]. It has been associated with dysregulation and overactivation of the alternative complement pathway due to genetic defects or the presence of autoantibodies that lead to vascular endothelial damage. Several triggering factors including infections, medications, autoimmune disorders, transplantation, pregnancy, and metabolic abnormalities have been linked to aHUS pathogenesis. The link between TMA or aHUS and *influenza* viruses, *Mycoplasma pneumoniae,* enteropathogenic *Escherichia coli* (EPEC), and *Helicobacter pylori* has been previously reported in the literature [3, 4, 5, 6]. We describe a case of aHUS triggered by *Influenza A* infection in the presence of other possible bacterial triggers, to raise awareness in the pediatric community pointing out that any infection may act as a trigger in aHUS pathogenesis. 

## Case report 

A 12-year-old boy was transferred to our department from a rural hospital with a 4-day history of fever, cough, dark-colored urine, vomiting, and weight loss without other gastrointestinal symptoms. His personal and family history were unremarkable, and his immunizations were up to date. He denied any recent travel or exposure to contaminated food or medication. Upon admission, he appeared unwell, with normal vital signs but jaundiced, with generalized abdominal pain, petechial rash on both legs and oral mucosa, hepatomegaly, and splenomegaly. Blood, urine, stool, and respiratory (upper and lower) samples were collected, and intravenous fluids and antibiotic treatment with ampicillin/sulbactam were started. Vital signs, including blood pressure control and diuresis by a bladder catheter, were monitored. 

Laboratory tests (Table 1) showed hemolytic anemia (decreased hemoglobin 10.6 g/dL, decreased haptoglobin 9.15 mg/dL, increased lactate dehydrogenase 1,765 U/L with negative direct Coombs test), severe thrombocytopenia (platelet count 12,000 μL), moderate lymphopenia (lymphocytes 670 μL), and elevated levels of indirect bilirubin 2.69 mg/dL. Plasma creatinine was 1.9 mg/dL, and blood urea was 135 mg/dL. Admission chest X-ray was normal. With these findings TMA was suspected, and the patient was transferred to our pediatric department. Coagulation studies showed an elevated D-Dimer concentration of 4,260 ng/mL. Peripheral blood smear revealed 4.6% schistocytes. Inflammation markers (C-reactive protein and erythrocyte sedimentation rate) were negative. In addition, protein elevation (100 mg/dL) and red blood cells (12 – 14 μL) were detected in a urine sample. In addition, glomerular filtration rate (GFR) was 38 mL/min/1.73m^2^. 

With the clinical suspicion of HUS, fresh frozen plasma (FFP) infusion (10 mL/kg) was initiated. *Influenza A* virus infection was confirmed on a nasopharyngeal swab and lower respiratory sample by polymerase chain reaction (PCR). Treatment with oseltamivir (per os) was started. EPEC was detected on a stool sample by PCR. Serum antibodies for *Mycoplasma pneumoniae* (IgM and IgG) and *Helicobacter pylori* (IgA and IgG) were detected. Blood, urine, stool, and pharyngeal cultures were negative. The evaluation with PCR and culture for Shiga toxin-producing *Escherichia coli* was also negative. Abdomen ultrasonography and Doppler ultrasound of the renal, hepatic, and splenic veins were conducted confirming hepatomegaly and splenomegaly. Autoimmunity markers (antinuclear and anti-ds-DNA autoantibodies) were negative. 

Investigation of the alternative complement pathway (with plasma levels C3, C4, and C5b-9, and next-generation sequencing (NGS) for the genes *ADAMTS-13, C3, CD46, CFB, CFH, MMACHC, CFHR1, CFHR3, CFHR4, CFHR5, CFI, INF2, DGKE, THBD, *and *VTN*) was undertaken to detect any complement dysregulation. C3 and C4 plasma levels were normal (80.4 and 25.1 mg/dL, respectively) as was ADAMTS-13, which was also normal (0.52 U/mL, normal range 0.41 – 1.41 U/mL), excluding the diagnosis of thrombotic thrombocytopenic purpura (TTP), but C5b-9 complex level was elevated (404 ng/mL, upper normal limit 245 ng/mL), leading to the diagnosis of aHUS, possibly triggered by multiple pathogens. NGS was negative for genetic defects. 

Our patient received FFP for 3 consecutive days followed by clinical and laboratory improvement. After FFP infusion, hemoglobin, platelets, lactate dehydrogenase, and kidney function parameters gradually improved (Table 1). Urine parameters also gradually normalized. He received treatment with oseltamivir for 5 days, and antibiotic treatment for a total of 3 days because of the suspicion of respiratory bacterial infection and the bladder catheter insertion. The patient had fever and gastrointestinal symptoms for a total of 5 days and dark-colored urine for 7 days. He was hospitalized for 10 days followed by regular follow-up visits to our pediatric nephrology outpatient clinic, in which he has remained asymptomatic up to the latest follow-up, 14 months after discharge. Since then, he has had two further infections (a viral upper respiratory infection and a viral gastroenteritis), which did not cause any further relapses or complications related to aHUS. 

## Discussion 

This is a rare case of *Influenza A-*triggered aHUS complicated by multiple infections at the same time playing a possible key role in aHUS pathogenesis. Infectious triggers have been correlated with aHUS [3, 4, 5, 6]. Our patient had a mild disease without a severe pattern as other TMA cases triggered by *Influenza A* infection [3, 7]. He also was treated with FFP, antibiotics, and antiviral therapy without any further need for step-up treatment either with dialysis, eculizumab, or ravulizumab for aHUS [1, 2]. Our patient’s condition raises many questions and possible hypotheses regarding the pathogenesis of aHUS. This was maybe a case of aHUS caused by complement dysregulation triggered by multiple pathogens. Unfortunately, there is no diagnostic test that confirms aHUS, and the diagnosis is one of exclusion [8]. *Influenza A* with neuraminidase production or direct endothelial cell damage leads to complement hyperactivity and finally aHUS [3]. Another hypothesis is that these triggers may have a direct role in vascular endothelial injury. 

Only 3 case reports have been reported that correlated HUS with *Mycoplasma pneumoniae* infection [4, 9, 10]. According to Valoti et al. [9], *Mycoplasma pneumoniae* may trigger anti-factor H antibody generation and aHUS in a genetically predisposed subject. Therefore, monitoring of anti-complement factor H antibodies in patients with glomerulonephritis after *Mycoplasma pneumoniae* infection could be recommended, especially in those with decreased C3 plasma levels. In our patient, C3 plasma levels were normal, and NGS was normal for mutations of complement genes [4, 9, 10]. Although genetic data are useful in the diagnosis of aHUS, the absence of detectable mutations in complement proteins does not rule out aHUS because genetic abnormalities are only identified in about half of patients [8]. 

According to Takatsuka et al. [6], *Helicobacter pylori* may stimulate the production of IL-12 and IL-8, leading to TMA due to the infection in the aplastic phase after bone marrow transplantation. The possible correlation between *Helicobacter pylori* and aHUS in previously healthy individuals remains unknown. This is the first report of positive *Helicobacter pylori* antibodies (IgA and IgG) possibly correlated with the pathogenesis of aHUS in a pediatric patient. 

Finally, EPEC has been associated with aHUS only in 1 adult patient [5]. Our patient did not have a history of diarrhea. So, the possible correlation between EPEC and aHUS remains unclear. 

In conclusion, aHUS is a rare and challenging type of TMA. Our patient had increased levels of C5b-9 complex, in combination with normal ADAMTS-13 levels and no history of diarrhea-transient complement activation triggered by infectious agents setting the diagnosis of “infection-mediated aHUS”. Early clinical suspicion and laboratory confirmation, result in early treatment and improvement of prognosis in patients with aHUS. 

## Authors’ contributions 

Nikolaos Gkiourtzis: concept and design of the study, interpretation of data, drafted the article, final approval of the version to be published. 

Paraskevi Panagopoulou: concept and design of the study, interpretation of data, critically revised the article, final approval of the version to be published. 

Kyriaki Papadopoulou-Legbelou: concept and design of the study, interpretation of data, critically revised the article, final approval of the version to be published. 

Sofia Chantavaridou: concept and design of the study, interpretation of data, drafted the article, final approval of the version to be published. 

Despoina Tramma: concept and design of the study, interpretation of data, critically revised the article, final approval of the version to be published. 

## Funding 

None. 

## Conflict of interest 

None to be declared. 

**Table 1.** Laboratory investigations during hospitalization.

Labs	Admission and initiation of treatment with FFP (day 4)	Day after treatment with FFP (day 7)	The day before the discharge (day 13)	First follow-up (day 20)	Second follow-up (day 50)
WBC (10^3^/μL)	5.63	4.99	7.48	5.96	5.08
Hb (g/dL)	10.6	9.2	10.6	10.8	13.7
PLT (10^3^/μL)	12	150	515	404	316
Schistocytes (%)	4.6	/	/	/	/
Cr (mg/dL)	1.9	1.01	0.71	0.58	0.56
Urea (mg/dL)	135	54	38	23	21
LDH (U/L)	1,765	638	134	197	154
Total bilirubin (mg/dL)	2.8	1.47	0.61	/	0.67

Cr = creatinine; Hb = hemoglobin; FFP = fresh frozen plasma; LDH = lactate dehydrogenase; PLT = platelets; WBC = white blood cells.
